# Six IL-8 gene polymorphisms and the entire cancer susceptibility according to a comprehensive analysis especially in prostate cancer

**DOI:** 10.1016/j.clinsp.2026.100862

**Published:** 2026-02-02

**Authors:** Xiao Zhang, Jian Sun, Xiqi Ding

**Affiliations:** Department of Urology, Affiliated Hospital of Jiangnan University, Wuxi, China

**Keywords:** Interleukin-8, Polymorphism, Risk, Prostate cancer, Meta-analysis

## Abstract

**Objectives:**

Interleukin-8 (IL-8) is a key cytokine regulating immunity and inflammation, and its genetic polymorphisms have been increasingly linked to cancer susceptibility. However, findings on the role of specific IL-8 polymorphisms across different cancers remain inconsistent, necessitating a comprehensive meta-analysis.

**Methods:**

The authors systematically searched Embase, PubMed, Chinese databases, Google Scholar, and Web of Science up to June 25, 2023. A total of 104 case-control studies (26,029 cases and 31,577 controls) were included in a high-powered meta-analysis. Summary Odds Ratios (ORs) with 95% Confidence Intervals (95% CIs) were calculated using Stata. Additionally, the authors performed an ELISA-based validation study to measure serum IL-8 levels in Prostate Cancer (PCa) patients grouped by genotype.

**Results:**

The +2767 polymorphism was associated with a significant reduction in overall cancer risk. The +781 polymorphism increased cancer risk specifically in Caucasian populations. In contrast, the -251 polymorphism demonstrated a strong pan-cancer risk association, particularly in Mixed/Asian ethnicities, gastric cancer, lung cancer, and hospital-based studies. Mechanistically, patients with the -251 AA/AC genotypes had elevated serum IL-8 levels, which were linked to more aggressive PCa pathogenesis.

**Conclusion:**

This pioneering meta-analysis identifies the IL-8 -251 polymorphism as a high-risk biomarker and +2767 as a protective variant. By functionally validating that the -251 risk allele promotes PCa progression via IL-8 overexpression, the present study establishes IL-8 genotyping as a potential tool for ethnic-specific risk stratification and prognostic prediction, with broad implications for precision oncology.

## Highlights

• +2767, +781, and −251 polymorphisms were associated with cancer risk.
• −251 variant may directly influence IL-8 expression in prostate cancer patients.
• These polymorphisms represent promising candidate biomarkers of cancer detection.

## Introduction

Rudolf Virchow reported a link between inflammation and cancer in the 19th century, based on an observation that leukocytes specifically invaded tumor tissue.^1^ Inflammation is an essential component governing all stages of tumor progression, including initiation, progression, malignant transformation, infiltration, and metastasis.^2^ Meanwhile, prolonged inflammation, lasting many years, causes severe DNA damage, which, in turn, can accelerate cancer progression.^3^ In addition, inflammation can also accelerate drug resistance; thus, combating drug resistance is a robust tool for cancer prevention, recovery, and intervention in cancer.^4^ Hence, an extensive elucidation of underlying inflammatory and cancer-related mechanisms may be highly beneficial to the establishment of new, robust, and specific cancer therapies.^2^^,^^5^

Interleukin-8 (IL-8) is an important inflammatory factor and is strongly upregulated during inflammation, and it has enhanced interacting capacities with receptors, compared to C-X-C motif chemokine relatives.^6^ Thus, it is believed to be the primary agent that recruits neutrophils to sites of inflammation. On the other hand, previous publications suggested that IL-8 plays an important role in tumor formation processes such as angiogenesis, tumorigenesis, tissue invasion, and metastasis.^7^ Moreover, the IL-18 was initially identified as a protein that induces Interferon-γ (IFNγ) production, which is an ambiguous role of the immune system in cancer progression.^8^^,^^9^

Given its critical roles in cancer development and polymorphisms may influence the expression of IL-8, for example, the −251T allele had 2- to 5-times stronger transcriptional activity than the −251A allele^10^^,^^11^; + 781C/T polymorphism may enhance gene transcription and regulation to influence IL-8 gene expression,^12^ numerous studies reported significant correlations between IL-8 polymorphisms and augmented cancer susceptibility in the recent decade.^13^^,^^14^ Despite several meta-analyses about associations between IL-8 polymorphisms and cancer risks being reported, the link still remains unclear. Hence, it is necessary to re-analyze to establish an evidence-based conclusion regarding IL-8 polymorphisms and cancer risk. In addition, the authors evaluated the association between IL-8 − 251 Polymorphism and PCa risk.

## Materials and method

### Screening and assessment of relevant investigations

The authors retrieved relevant information from multiple scientific databases (Embase, PubMed, Chinese database, Google Scholar, and Web of Science) from the date of establishment till Jun 25, 2023. The employed search terms were ‘Interleukin-8′ or ‘IL-8′, and ‘polymorphism’, and ‘cancer’ or ‘tumor’. Collectively, the authors identified 973 relevant scientific papers, among which 104 fit our strict inclusion and exclusion criteria. Additionally, the authors also manually screened references of relevant review articles.

### Inclusion and exclusion criteria

The following research articles were included: (a) Examined cancer association with IL-8 gene polymorphisms; (b) Case-control study; (c) Case/control groups with adequate genotype frequency; (d) Study based on the Hardy-Weinberg Equilibrium (HWE). The following research articles were excluded from analysis: (a) Absence of control, (b) No available data on genotype frequency, and (c) duplicated case-control groups.

### Data retrieval

The authors extracted the following information from the selected articles: last name of first author, publication year, corresponding author's country of residence, cancer category, ethnicity, control source, number of each genotype of cases and controls, HWE for control, and genotyping method.

### Statistical analysis

Study subjects were classified as Asian, Caucasian, African, and Mixed population. The control subgroup source was stratified into Hospital-Based (HB) and Population-Based (PB). The genotype methods were classified into Taqman, PCR-RFLP, ARMS-PCR, PCR, and AS-PCR. To determine genotype frequencies among cases and Healthy Controls (HCs), the authors used the Odds Ratios (OR) with 95 % Confidence Intervals (95 % CI) to evaluated the associations among the IL-8 gene polymorphisms and the whole cancer risk. Besides, the overall OR was assessed with the *Z*-test.^15^ In the presence of significant heterogeneity by *Q*-test (*p* > 0.05), the authors employed the random-effects model; otherwise, the fixed-effects model was used.^16^^,^^17^ Five models (allelic contrast, homozygote comparison, dominant genetic model, heterozygote comparison, and recessive genetic model) were assessed to the correlation between various IL-8 gene polymorphisms and the entire cancer risk. The HWE from control groups was computed using the Pearson Chi-Square test. The Egger’s regression test and Begg’s funnel plots were employed for publication bias detection.^18^ All data analyses were conducted using Stata V11.2 (StataCorp LP, College Station, TX).

### Participant demographics

Herein, 100 patients, who were newly diagnosed of (PCa) between Jun 1th, 2017 and Nov 1th, 2022, were recruited from the Affiliated Hospital of Jiangnan University. All diagnoses were based on histological examinations. HCs were age-matched to cancer patients, prostate-specific antigen < 4.0 ng/mL, negative results from digital rectal examination, and no family history of prostate cancer. This work received ethical approval from the Affiliated Hospital of Jiangnan University and obtained informed consent from all participants prior to the initiation of the study.

### Enzyme-Linked immunosorbent assay (ELISA)

Following standard blood collection without anticoagulants, the serum IL-8 content was measured using the ELISA kit (Abcam Co. Ltd.), as described in a previous publication.^19^ For further information, please refer to the following website: (https://www.abcam.cn/products/elisa/human-il-8-elisa-kit-ab214030.html).

## Results

### Meta-analysis study selection and characteristics

**Fig. 1:**
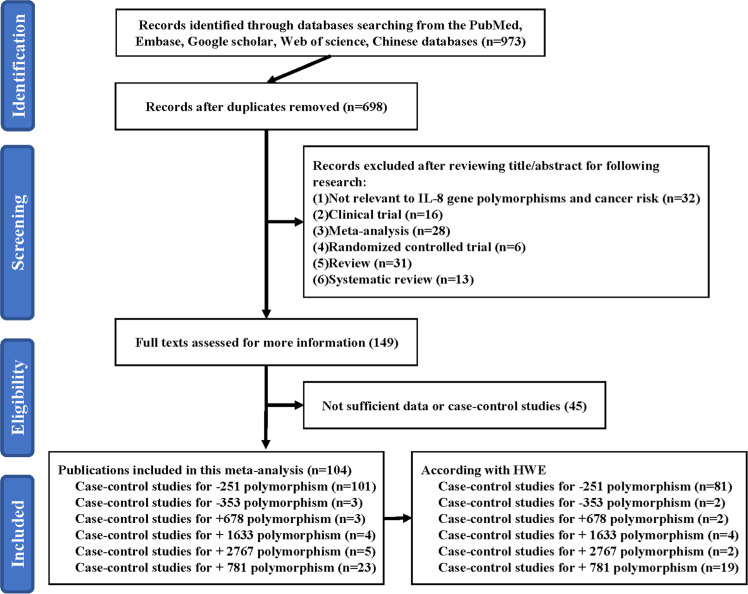
A schematic of the six IL-8 gene polymorphisms screening process.

**Fig. 2:**
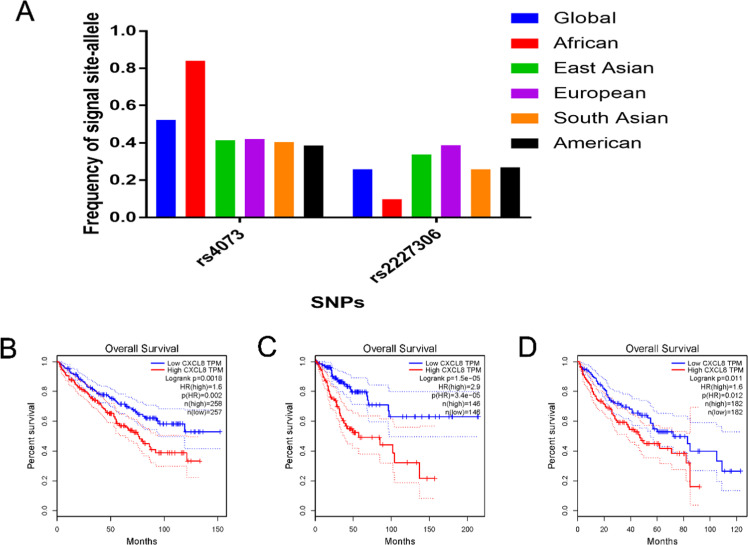
(A) The minor-allele (mutant-allele) MAF for IL-8 polymorphisms (rs4073 -251 site, rs2227306-+781 site) from the 1000 Genomes online database. (B) The different expression of IL-8 and the relative relationship for overall survival in Kidney Renal Clear Cell Carcinoma (KIRC). (C) The different expression of IL-8 and the relative relationship for overall survival in Cervical Squamous Cell Carcinoma and Endocervical Adenocarcinoma (CESC). (D) The different expression of IL-8 and the relative relationship for overall survival in Liver Hepatocellular Carcinoma (LICH).

In all, the authors selected 973 eligible articles using the aforementioned search terms, and only 104 (retrieved on 25 Jun 2023) fit our strict inclusion and exclusion criteria. In short, 698 duplications were eliminated. The authors identified 28 meta-analyses, 32 other gene polymorphisms, 16 clinical trials, 6 randomized controlled trials, 31 reviews, and 13 systematic reviews. Among the remaining 149 articles, 45 lacked complete genotyping information, which were excluded as well (Fig. 1). Supplemental Table 1 summarizes all characteristics of included studies (namely, first author information, ethnicity, year of publication, country, cancer category, HC and case quantity, genotyping methodology, HWE, and control sources). Among these, the authors identified 2 publications on −353 polymorphism, 2 on +678 polymorphism, 2 on +2767 polymorphism, 4 on +1633 polymorphism, 19 on +781 polymorphism, and 81 on −251 polymorphism. All analyzed studies were based on HWE. In addition, the authors also get big data from different websites to analyze IL-8 and its polymorphisms. First, the authors acquired the Minor Allele Frequency (MAF) of +781 and −251 polymorphisms in six major global populations analyzed from 1000 Genomes Browser (https://www.ncbi.nlm.nih.gov/snp/rs2227306 or rs4073) (Fig. 2A). Second, through the GEPIA website (http://gepia.cancer-pku.cn/),^20^ the authors analyzed all kinds of cancer, three types of cancer (kidney renal clear cell carcinoma, cervical squamous cell carcinoma and endocervical adenocarcinoma, liver hepatocellular carcinoma) show the significant results between high/low expression of IL-8 and overall survival, which all have the same tread that high expression of IL-8 had a short overall survival (Fig. 2B‒D).

### Meta-analysis

Supplemental Table 2 lists the relationship between six distinct IL-8 polymorphisms and the overall cancer risk. No association was detected among IL-8 − 353, +678, +1633 polymorphisms and cancer risk.

### +2767 A/G site

**Fig. 3:**
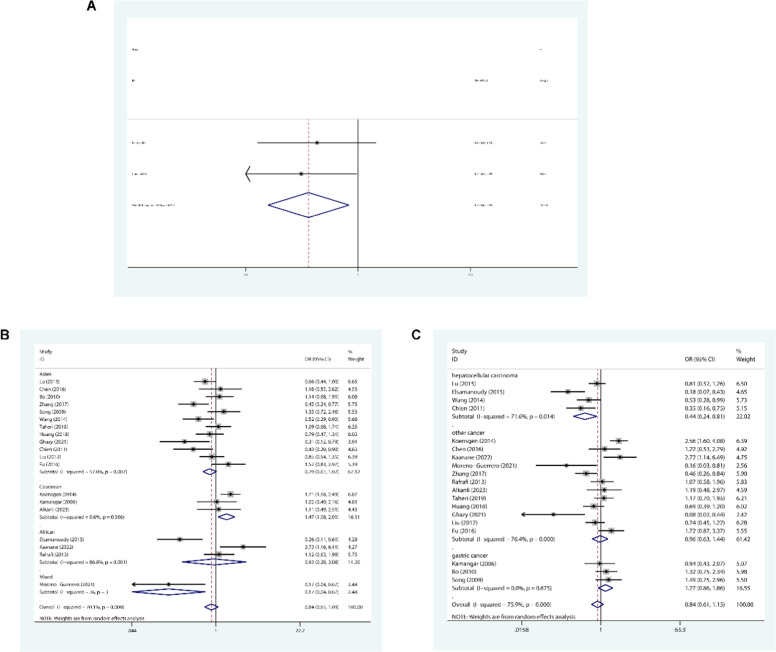
Forest plot illustrating correlation between two IL-8 gene polymorphisms and cancer risk. (A) The authors observed reduced relation between the +2767 site (T-vs. C-allele) and overall cancer risk; (B) The authors observed enhanced relation between the +781 site (TT vs. TC+CC) and cancer risk within the Caucasian subgroup; (C) The authors observed reduced relation between the +781 site (TT vs. CC) and hepatocellular carcinoma in the cancer category subgroup.

In case of the +2767 site, the authors observed reduced association with cancer risk in three genetic models (A-allele vs. G-allele: OR = 0.766, 95 % CI: 0.616‒0.953, *p* = 0.017; AA vs. GG: OR = 0.592, 95 % CI: 0.378‒0.928, *p* = 0.022; AA vs. AG+GG: OR = 0.652, 95 % CI: 0.429‒0.991, *p* = 0.045, Fig. 3A).

### +781 T/C site

The authors revealed enhanced association among Caucasians for the +781 T/C polymorphism (TT vs. TC+CC: OR = 1.472, 95 % CI: 1.078‒2.009, *p* = 0.015, Fig. 3B), but reduced relation in hepatocellular carcinoma (TT vs. CC: OR = 0.437, 95 % CI: 0.235‒0.812, *p* = 0.009, Fig. 3C; TT vs. TC+CC: OR = 0.499, 95 % CI: 0.349‒0.711, *p* < 0.001).

### -251A/G site

**Fig. 4:**
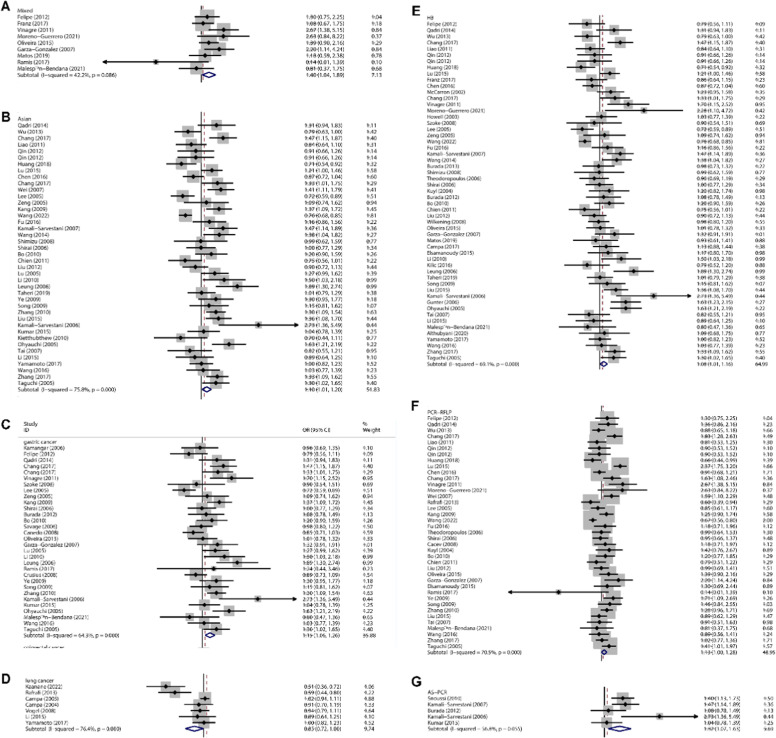
Forest plot illustrating correlation between the IL-8 -251 gene polymorphism and overall cancer risk. (A) The authors observed enhanced relation between the -251 site (AC vs. CC) and cancer risk among the Mixed population. (B) The authors observed enhanced relation between the -251 site (A- vs. C-allele) and cancer risk among the Asian population. (C) The authors observed enhanced relation between the -251 site (A- vs. C-allele) and gastric cancer among the ethnic subgroup. (D) The authors observed reduced relation between the -251 site (A- vs. C-allele) and lung cancer in the ethnic subgroup. (E) The authors observed enhanced relation between the -251 site (A- vs. C-allele) and HB control source. (F) The authors observed enhanced relation between the -251 site (AC vs. CC) and the PCR-RFLP genotype subgroup. (G) The authors observed enhanced relation between the -251 site (A- vs. C-allele) and the AS-PCR genotype subgroup.

There were increased associations between the -251 polymorphism and total cancer risk (for example: A-allele vs. C-allele: OR = 1.055, 95 % CI: 1.000‒1.112, *p* = 0.049) among Mixed populations (AG vs. CC: OR = 1.402, 95 % CI: 1.041‒1.889, *p* = 0.026, Fig. 4A), Asians (for example: A- vs. C-allele: OR = 1.102, 95 % CI: 1.014‒1.197, *p* = 0.022, Fig. 4B). In addition, in the ethnicity subgroup, similar positive results were found in gastric cancer (for example: A- vs. C-allele: OR = 1.155, 95 % CI: 1.057‒1.262, *p* = 0.002, Fig. 4C). However, reduced relation was present between -251 polymorphism and lung cancer risk (A- vs. C-allele: OR = 0.845, 95 % CI: 0.717‒0.996, *p* = 0.045, Fig. 4D). In the source of control, significant association was observed in HB (for example: A- vs. C-allele: OR = 1.078, 95 % CI: 1.005‒1.155, *p* = 0.035, Fig. 4E). Finally, in the subgroup of genotype method, increased relationship was detected in the PCR-RFLP (AG vs. CC: OR = 1.131, 95 % CI: 1.000‒1.278, *p* = 0.050, Fig. 4F), and AS-PCR methods (A- vs. C-allele: OR = 1.323, 95 % CI: 1.074‒1.629, *p* = 0.008, Fig. 4G).

### Publication sensitivity and bias analysis

**Fig. 5:**
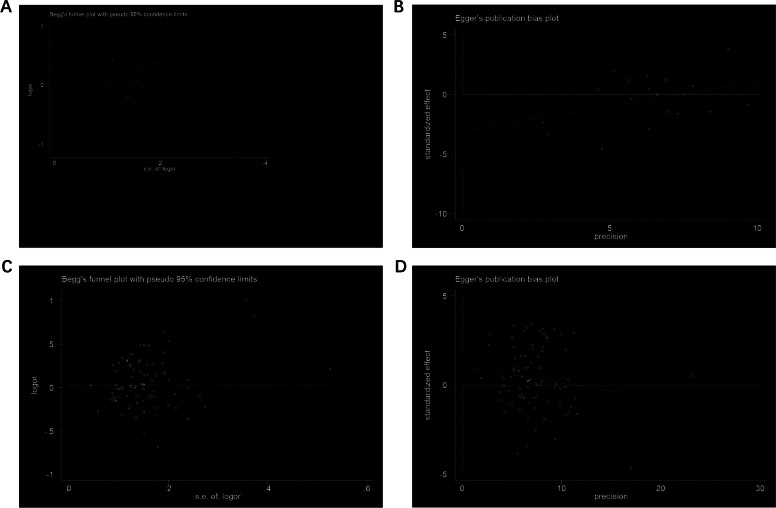
Publication bias analysis. (A) Begg’s funnel plot for publication bias (T-allele vs. C-allele) for +781 site. (B) The plot of Egger’s publishing bias (T-allele vs. C-allele) for +781 site. (C) Begg’s funnel plot for publication bias (A-allele vs. C-allele) for -251 site. (D) The plot of Egger’s publishing bias (A-allele vs. C-allele) for -251 site.

To assess publication bias, Begg’s and Egger’s tests were used to evaluate. Finally, there was no indication about publication bias from both tests in both -251 and +781 polymorphisms (for example, in allelic contrast, Fig. 5).

### Serum IL-8 expression in PCa patients

**Fig. 6:**
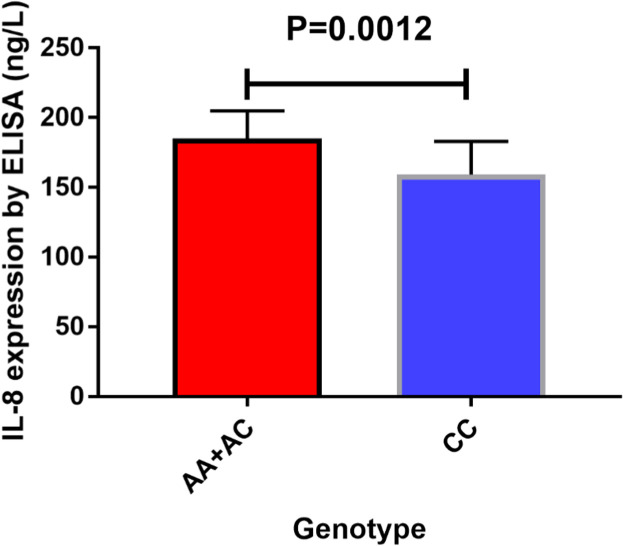
Circulating IL-8 content in the -251 genotype of (PCa) using average values (horizontal lines, average values). Circulating IL-8 content in (PCa) patients with the AA+CC genotype was substantially elevated, compared to those carrying the CC genotypes.

Herein, the authors collected 100 serum samples from (PCa) patients, specifically representing various genotypes of the IL-8 -251 variant. Based on our observation, the AA, AC, and CC genotype distribution was 19, 27, and 54, respectively. Additionally, the authors also assessed the serum IL-8 expression using ELISA. In particular, the authors examined the AA/AC and CC genotypes, our analysis revealed that the circulating IL-8 content among (PCa) patients with the AA/AC genotypes were substantially elevated, compared to those with the CC genotypes (*p* = 0.0012, Fig. 6).

## Discussion

Long-term inflammation or autoimmune diseases are intricately linked to numerous human diseases, including asthma, rheumatoid arthritis, systemic lupus erythematosus, inflammatory bowel disease, and cancer.^21^ Tumor-related inflammation is the seventh major characteristic of all cancers.^22^ In the tumor immune microenvironment, innate immune cells, particularly monocytes and adaptive immune cells (e.g., T-lymphocytes), associate with or release several inflammatory regulators like tumor necrosis factor and IL-8, which transmit abnormal signals from tumors.^23^^,^^24^

Multiple meta-analyses reported on the strong connection between IL-8 gene polymorphisms and cancer. For example, Zhang et al. comprised 22 case-control investigations, involving 4259 cancer cases, 7006 HCs, and 5 polymorphisms (rs2227307, rs2227306, +678T/C, rs1126647, and +1633C/T), and revealed that only the rs2227306 polymorphism was protective against hepatocellular carcinoma development.^14^ In addition, Wang et al. analyzed 12,917 cases with varying cancer categories and 17,689 HCs from 47 publications, and demonstrated that the -251 polymorphism was strongly related to enhanced cancer risk.^25^ However, despite multiple investigations examining this potential relationship, the overall conclusion remains unclear.

Herein, the authors employed 104 case-control studies, involving 26,029 cancer incidences and 31,577 HCs. The authors revealed no discernible differences in tumor risk based on three IL-8 polymorphisms (-353, +678, +1633) in our genetic model. Meanwhile, the +2767 polymorphism protected against cancer susceptibility. However, the +781 polymorphism exhibited differing relations with cancer risk. Alternately, the authors observed an increased relationship with Caucasians and a decreased relationship with hepatocellular carcinoma incidence. The authors observed comparable results in the -251 polymorphism, with reduced correlation with lung cancer. Conversely, there had enhanced association in the whole samples, ethnicity (Mixed and Asian population), gastric cancer, HB, PCR-RFLP, and AS-PCR subgroups. The potential reasons may be as follows: a given gene polymorphism may serve varying functions in distinct cancer categories, likely due to differences in cancer pathology. Additionally, the same gene and its polymorphisms may be responsible for several physiological activities. Inter-ethnic genetic and environmental differences may be another factor, along with linkage disequilibrium patterns, that regulate the function of differential gene polymorphisms’ function among different patient populations. The two aforementioned polymorphisms (-251 and +781) have great potential as clinical bioindicators of cancer risk. For instance, someone carrying an A-allele or the AA genotype may possess an enhanced likelihood of developing (PCa). Thus, early detection, intervention, and prevention may potentially reduce cancer risk and enhance patient outcome.

Some limitations of our investigation need to be considered. Firstly, this study did not discuss the effect of synergistic environment-gene or gene-gene interaction; thus, this needs to be addressed in the future. Secondly, the carcinogenic effect of a single factor within a given population may be concealed by other stronger carcinogenic agents, which may exert synergistic or antagonistic effects. Thirdly, it is imperative to develop an extensive research model or database, with a vast patient population, influencing factors elimination, and enhanced ethnic group separation for homogeneity minimization before conducting a systematic multi-factorial analysis to alter the detection, diagnosis, and treatment of distinct patient populations. Fourthly, the authors recommend increasing the sample size for the -353, +678, +1633, and +2767 polymorphisms in future research. Fifthly, there needs to be extensive studies on the various functions (e.g., luciferase assays, CRISPR edits, prospective validation or therapeutic implications, correlation between different genotypes and IL-8 expression using tumor staining (IHC) in patient samples, immunotherapy response (e.g., IL-8 blockade in high-risk genotypes), proposing a genetic risk score combining IL-8 SNPs with other markers) of the six IL-8 gene polymorphisms based on different genotypes to explain how these SNPs modulate IL-8 expression and cancer pathways.

Finally, the research prospects of the present study are put forward. The future of functional SNPs (fSNPs) research lies in a synergistic approach that couples large-scale, rigorous clinical validation with deep mechanistic inquiry using cutting-edge technologies. Overcoming challenges related to ethnic diversity, polygenicity, and ethical considerations will be paramount. By firmly establishing the link between genotype and phenotype, fSNPs are poised to evolve from statistical associations into indispensable tools for risk stratification, companion diagnostics, and the development of novel therapeutics, thereby fulfilling the core promise of precision medicine.

## Conclusion

Herein, the authors demonstrated that the three IL-8 gene polymorphisms, including +2767, +781, and -251 were essential genetic factors that regulate cancer susceptibility. In addition, -251 polymorphism may be considered as a warning factor for PCa detection, which will be helpful for supplementing existing diagnostic techniques, ultimately improving the early diagnosis and treatment of PCa, and improving the overall survival time and quality of life of patients. The authors warrant additional extensive investigations on these polymorphisms in the laboratory and clinic.

## Ethics statement

Ethical review and approval were not required for this animal study, in accordance with the local legislation and institutional requirements.

## Availability of data and materials

The datasets used and/or analyzed during the present study are available from the corresponding author upon reasonable request.

## Authors’ contributions

Xiao Zhang conceived and designed the study. Jian Sun collected data. Jian Sun analyzed the data. Xiqi Ding drafted the manuscript. All authors have reviewed and approved the final version of the manuscript prior to submission.

## Funding

This study was supported by “Key Medical Technology and Key Platform Challenge Projects” from the Affiliated Jiangnan University Hospital.

## Declaration of competing interest

The authors declare no conflicts of interest.

## Appendix Supplementary materials

Image, application 1
