# Supplementary material for: Six IL-8 gene polymorphisms and the entire cancer susceptibility according to a comprehensive analysis especially in prostate cancer
Source: Clinics (Sao Paulo). 2026 Feb 2;81:100862. doi: 10.1016/j.clinsp.2026.100862 (PMC12886085; doi:10.1016/j.clinsp.2026.100862)
Supplement: Supplementary file 1 [file mmc1.docx]

**CLINICS-D-24-00813_Supplementary Material**

**Supplemental Table 1** Characteristics of included studies about polymorphisms in IL-8 gene polymorphisms and cancer risk.

| **Author** | **Year** | **Country** | **Ethnicity** | **Cancer type** | **Case** | **Control** | **SOC** | **HWE** | **Genotype** |
| --- | --- | --- | --- | --- | --- | --- | --- | --- | --- |
| -251 site |  |  |  |  |  |  |  |  |  |
| Smith | 2004 | UK | Caucasian | BRC | 119 | 235 | PB | 0.131 | ARMS-PCR |
| Zhang | 2017 | China | Asian | BRC | 442 | 447 | HB | 0.948 | PCR-RFLP |
| Kamali-Sarvestani | 2007 | Iran | Asian | BRC | 257 | 233 | HB | 0.26 | ASO-PCR |
| Snoussi | 2010 | Tunisia | African | BRC | 409 | 301 | PB | 0.173 | AS-PCR |
| Wang | 2022 | China | Asian | BRC | 1232 | 1232 | HB | 0.231 | PCR-RFLP |
| Althubyani | 2020 | Eygypt | African | COC | 70 | 70 | HB | 0.932 | TaqMan |
| Burada | 2013 | Romania | Caucasian | COC | 144 | 233 | HB | 0.291 | TaqMan |
| Theodoropoulos | 2006 | Greece | Caucasian | COC | 222 | 196 | HB | 0.327 | PCR-RFLP |
| Basavaraju | 2015 | USA | Caucasian | COC | 388 | 491 | PB | 0.711 | TaqMan |
| Tsilidis | 2009 | USA | Caucasian | COC | 205 | 362 | PB | 0.058 | TaqMan |
| Gunter | 2006 | Italy | Caucasian | COC | 205 | 191 | HB | 0.84 | TaqMan |
| Wilkening | 2008 | Sweden | Caucasian | COC | 300 | 580 | HB | 0.476 | TaqMan |
| Vogel | 2007 | Denmark | Caucasian | COC | 355 | 753 | PB | 0.627 | PCR-CE-SSCP |
| Malespín-Bendana | 2021 | Costa Rica | Mixed | GC | 46 | 81 | HB | 0.907 | PCR-RFLP |
| Kamali-Sarvestani | 2006 | Iran | Asian | GC | 19 | 153 | HB | 0.797 | ASO-PCR |
| Qadri | 2014 | India | Asian | GC | 130 | 200 | HB | 0.066 | PCR-RFLP |
| Kamangar | 2006 | USA | Caucasian | GC | 112 | 207 | PB | 0.055 | TaqMan |
| Felipe | 2012 | Brasil | Mixed | GC | 104 | 196 | HB | 0.065 | PCR-RFLP |
| Garza-Gonzalez | 2007 | USA | Mixed | GC | 78 | 207 | HB | 0.492 | PCR-RFLP |
| Ye | 2009 | Korea | Asian | GC | 153 | 206 | PB | 0.72 | PCR-RFLP |
| Shirai | 2006 | Japan | Asian | GC | 181 | 468 | HB | 0.343 | PCR-RFLP |
| Song | 2009 | China | Asian | GC | 125 | 140 | HB | 0.72 | PCR-RFLP |
| Burada | 2012 | Craiova | Caucasian | GC | 105 | 242 | HB | 0.386 | AS-PCR |
| Vinagre | 2011 | Brasil | Mixed | GC | 102 | 103 | HB | 0.15 | PCR-RFLP |
| Wang | 2016 | China | Asian | GC | 132 | 296 | HB | 0.946 | PCR-RFLP |
| Zeng | 2005 | China | Asian | GC | 104 | 94 | HB | 0.212 | PCR-RDB |
| Chang | 2017 | Korea | Asian | GC | 283 | 176 | HB | 0.136 | PCR-RFLP |
| Chang | 2017 | Korea | Asian | GC | 283 | 284 | HB | 0.082 | PCR-RFLP |
| Bo | 2010 | China | Asian | GC | 208 | 190 | HB | 0.389 | PCR-RFLP |
| Taguchi | 2005 | Japan | Asian | GC | 396 | 252 | HB | 0.994 | PCR-RFLP |
| Oliveira | 2015 | Brazil | Mixed | GC | 240 | 207 | HB | 0.488 | PCR-RFLP |
| Kumar | 2015 | India | Asian | GC | 200 | 182 | PB | 0.801 | ASO-PCR |
| Kang | 2009 | Korea | Asian | GC | 334 | 322 | PB | 0.226 | PCR-RFLP |
| Canedo | 2008 | France | Caucasian | GC | 333 | 693 | PB | 0.459 | TaqMan |
| Lu | 2005 | China | Asian | GC | 250 | 300 | PB | 0.516 | PCR-DHPLC |
| Lee | 2005 | China | Asian | GC | 461 | 303 | HB | 0.184 | PCR-RFLP |
| Savage | 2006 | USA | Caucasian | GC | 287 | 428 | PB | 0.391 | TaqMan |
| Zhang | 2010 | China | Asian | GC | 519 | 504 | PB | 0.754 | PCR-RFLP |
| Li | 2010 | China | Asian | GC | 101 | 137 | HB | 0.579 | PCR-DHPLC |
| Ohyauchi | 2005 | Japan | Asian | GC | 212 | 244 | HB | 0.847 | DS |
| Crusius | 2008 | France | Caucasian | GC | 236 | 1139 | PB | 0.705 | Real-Time PCR |
| Leung | 2006 | China | Asian | GC | 83 | 179 | HB | 0.638 | TaqMan |
| Szoke | 2008 | Hungary | Caucasian | GC | 35 | 168 | HB | 0.165 | ARMS-PCR |
| Ramis | 2017 | Brazil | Mixed | GC | 9 | 38 | PB | 0.691 | PCR-RFLP |
| Fu | 2016 | China | Asian | Glioma | 127 | 284 | HB | 0.251 | PCR-RFLP |
| Liu | 2015 | China | Asian | Glioma | 300 | 300 | HB | 0.772 | PCR-RFLP |
| Chien | 2011 | China | Asian | HC | 131 | 340 | HB | 0.445 | PCR-RFLP |
| Wang | 2014 | China | Asian | HC | 205 | 208 | HB | 0.266 | PCR-RFLP/PCR-SSP |
| Liao | 2011 | China | Asian | HC | 390 | 150 | HB | 0.104 | PCR-RFLP |
| Elsamanoudy | 2015 | Egypt | African | HC | 112 | 105 | HB | 0.551 | PCR-RFLP |
| Qin | 2012 | China | Asian | HC | 150 | 150 | HB | 0.104 | PCR-RFLP |
| Lu | 2015 | China | Asian | HC | 454 | 446 | HB | 0.115 | PCR-RFLP |
| Rafrafi | 2013 | Tunisia | African | LC | 170 | 225 | PB | 0.181 | PCR-RFLP |
| Yamamoto | 2017 | Japan | Asian | LC | 462 | 379 | HB | 0.939 | TaqMan |
| Campa | 2004 | Norway | Caucasian | LC | 239 | 210 | PB | 0.317 | TaqMan |
| Kaanane | 2022 | Morocco | African | LC | 150 | 150 | PB | 0.169 | TaqMan |
| Campa | 2005 | Germany | Caucasian | LC | 2144 | 2116 | PB | 0.203 | TaqMan |
| Vogel | 2008 | Denmark | Caucasian | LC | 403 | 744 | PB | 0.672 | PCR-FLP |
| Li | 2015 | China | Asian | LC | 132 | 150 | HB | 0.894 | PCR-HRM |
| Tai | 2007 | China | Asian | NC | 105 | 109 | HB | 0.886 | PCR-RFLP |
| Huang | 2018 | China | Asian | NC | 176 | 352 | HB | 0.109 | PCR-RFLP |
| Nasr | 2007 | Tunisia | African | NC | 160 | 169 | PB | 0.349 | PCR-SSP |
| Wei | 2007 | China | Asian | NC | 280 | 290 | PB | 0.164 | PCR-RFLP |
| Matos | 2019 | Brazil | Mixed | OC | 66 | 130 | HB | 0.493 | PCR |
| Qin | 2012 | China | Asian | OC | 150 | 150 | HB | 0.104 | PCR-RFLP |
| Liu | 2012 | China | Asian | OC | 270 | 350 | HB | 0.454 | PCR-RFLP |
| Campa | 2017 | Germany | Caucasian | OC | 153 | 725 | HB | 0.524 | TaqMan |
| Shimizu | 2008 | Japan | Asian | OC | 69 | 91 | HB | 0.296 | PCR-FLP |
| Kietthubthew | 2010 | Thailand | Asian | OC | 63 | 99 | PB | 0.813 | TaqMan |
| Moreno-Guerrero | 2021 | Mexico | Mixed | Neuroblastoma | 27 | 38 | HB | 0.152 | PCR-RFLP |
| Kilic | 2016 | Turkey | Caucasian | Thyroid cancer | 101 | 109 | HB | 0.586 | PCR |
| Wu | 2013 | China | Asian | Urothelial carcinoma | 300 | 594 | HB | 0.075 | PCR-RFLP |
| Kuyl | 2004 | Netherlands | Caucasian | Kaposi’s sarcoma | 84 | 153 | HB | 0.382 | PCR-RFLP |
| Howell | 2003 | UK | Caucasian | Melanoma | 142 | 233 | HB | 0.16 | ARMS–PCR |
| Cacev | 2008 | Croatia | Caucasian | Colon cancer | 160 | 160 | PB | 0.346 | PCR-RFLP |
| Franz | 2017 | Brazil | Mixed | PC | 175 | 185 | HB | 0.127 | PCR-SSP |
| Chen | 2016 | China | Asian | PC | 439 | 524 | HB | 0.129 | PCR-RFLP |
| Taheri | 2019 | Iran | Asian | PC | 355 | 200 | HB | 0.689 | ARMS-PCR |
| Yang | 2006 | USA | Caucasian | PC | 520 | 418 | PB | 0.168 | ht-SNP |
| McCarron | 2002 | UK | Caucasian | PC | 238 | 235 | HB | 0.131 | PCR |
| Michaud | 2006 | USA | Caucasian | PC | 484 | 613 | PB | 0.777 | PCR |
| -353 site |  |  |  |  |  |  |  |  |  |
| Wei | 2007 | China | Asian | NC | 280 | 290 | PB | 0.406 | PCR-RFLP |
| Wang | 2014 | China | Asian | HC | 205 | 208 | HB | 0.474 | PCR-RFLP/PCR-SSP |
| +678 site |  |  |  |  |  |  |  |  |  |
| Wei | 2007 | China | Asian | NC | 280 | 290 | PB | 0.064 | PCR-RFLP |
| Wang | 2014 | China | Asian | HC | 205 | 208 | HB | 0.161 | AS-PCR |
| +1633 site |  |  |  |  |  |  |  |  |  |
| Chien | 2011 | China | Asian | HC | 131 | 340 | HB | 0.562 | PCR-RFLP |
| Liu | 2012 | China | Asian | OC | 270 | 350 | HB | 0.569 | PCR-RFLP |
| Koensgen | 2014 | Germany | Caucasian | Ovarian cancer | 246 | 62 | HB | 0.865 | PCR-RFLP |
| Huang | 2018 | China | Asian | NC | 176 | 352 | HB | 0.109 | PCR-RFLP |
| +2767 site |  |  |  |  |  |  |  |  |  |
| Chien | 2011 | China | Asian | HC | 131 | 340 | HB | 0.392 | PCR-RFLP |
| Hsieh | 2007 | China | Asian | Leiomyoma | 162 | 156 | HB | 0.078 | PCR-RFLP |
| +781 site |  |  |  |  |  |  |  |  |  |
| Kamangar | 2006 | USA | Caucasian | GC | 111 | 208 | PB | 0.158 | TaqMan |
| Bo | 2010 | China | Asian | GC | 208 | 190 | HB | 0.225 | PCR-RFLP |
| Chien | 2011 | China | Asian | HC | 131 | 340 | HB | 0.776 | PCR-RFLP |
| Liu | 2012 | China | Asian | OC | 270 | 350 | HB | 0.781 | PCR-RFLP |
| Rafrafi | 2013 | Tunisia | African | LC | 170 | 225 | PB | 0.329 | PCR-RFLP |
| Wang | 2014 | China | Asian | HC | 205 | 208 | HB | 0.549 | PCR-RFLP/PCR-SSP |
| Koensgen | 2014 | Germany | Caucasian | Ovarian cancer | 267 | 426 | HB | 0.1 | PCR-RFLP |
| Chen | 2016 | China | Asian | Osteosarcoma | 190 | 190 | HB | 0.116 | PCR-RFLP |
| Taheri | 2019 | Iran | Asian | PC | 355 | 200 | HB | 0.639 | ARMS-PCR |
| Kaanane | 2022 | Morocco | African | LC | 150 | 150 | PB | 0.307 | TaqMan |
| Alkanli | 2023 | Turkey | Caucasian | Bladder cancer | 88 | 89 | HB | 0.608 | PCR-RFLP |
| Moreno-Guerrero | 2021 | Mexico | Mixed | Neuroblastoma | 27 | 38 | HB | 0.313 | PCR-RFLP |
| Song | 2009 | China | Asian | GC | 125 | 140 | HB | 0.48 | PCR-RFLP |
| Fu | 2016 | China | Asian | Glioma | 127 | 284 | HB | 0.788 | PCR-RFLP |
| Zhang | 2017 | China | Asian | BRC | 442 | 447 | HB | 0.327 | PCR-RFLP |
| Huang | 2018 | China | Asian | NC | 176 | 352 | HB | 0.671 | PCR-RFLP |
| Ghazy | 2021 | Saudi Arabia | Asian | PC | 40 | 40 | HB | 0.673 | real-time PCR |
| Elsamanoudy | 2015 | Egypt | African | HC | 112 | 105 | HB | 0.178 | PCR-RFLP |
| Lu | 2015 | China | Asian | HC | 454 | 446 | HB | 0.062 | PCR-RFLP |

HB, Hospital-Based; PB, Population-Based; SOC, Source of Control; PCR-RFLP, Polymerase Chain Reaction followed by Restriction Fragment Length Polymorphism; SSP, Sequence Specific Primer; AS, Allele Specific Primer; ARMS, Amplification Refractory Mutation System; MALDI-TOF MS, Matrix-Assisted Laser Desorption/Ionization time of flight Mass Spectrometry; HRM, High Resolution Melt; DHPLC, Denaturing High Performance Liquid chromatography; CE-SSCP, Capillary Electrophoresis-Single Strand Conformation Polymorphism; HWE, Hardy-Weinberg Equilibrium of control group; BRC, Breast Cancer; COC, Colorectal Cancer; GC, Gastric Cancer; HC, Hepatocellular Carcinoma; LC, Lung Cancer; NC, Nasopharyngeal Carcinoma; OC, Oral Cancer; PC, Prostate Cancer.

**Supplemental Table 2** Stratified analyses of IL-8 genes common polymorphisms on cancer risk.

| **Variables** | **No** | **Case/Controls** | **M-allele vs. W-allele** | | **MM vs. WW** | | **MW vs. WW** | | **MM+MW vs. WW** | | **MM vs. MW+WW** | |
| --- | --- | --- | --- | --- | --- | --- | --- | --- | --- | --- | --- | --- |
|  |  |  | **OR (95% CI)** | | **OR (95% CI)** | | **OR (95% CI)** | | **OR (95% CI)** | | **OR (95% CI)** | |
|  |  |  | ***P*_h_** | **p** | ***P*_h_** | **p** | ***P*_h_** | **p** | ***P*_h_** | **p** | ***P*_h_** | **p** |
| IL-8 -353 | 2 | 485/498 | 1.192 (0.996‒1.427) | | 1.374 (0.954‒1.980) | | 1.257 (0.951‒1.661) | | 1.287 (0.991‒1.672) | | 1.205 (0.868‒1.675) | |
|  |  |  | 0.456 | 0.056 | 0.516 | 0.088 | 0.442 | 0.108 | 0.399 | 0.059 | 0.737 | 0.265 |
| IL-8 +678 | 2 | 485/498 | 1.025 (0.848‒1.239) | | 1.092 (0.738‒1.617) | | 0.966 (0.738‒1.266) | | 0.997 (0.776‒1.280) | | 1.109 (0.763‒1.612) | |
|  |  |  | 0.822 | 0.799 | 0.830 | 0.659 | 0.922 | 0.804 | 0.868 | 0.980 | 0.847 | 0.589 |
| IL-8 +2767 | 2 | 293/496 | 0.766 (0.616‒0.953) | | 0.592 (0.378‒0.928) | | 0.823 (0.596‒1.137) | | 0.754 (0.557‒1.020) | | 0.652 (0.429‒0.991) | |
|  |  |  | 0.704 | 0.017 | 0.829 | 0.022 | 0.443 | 0.238 | 0.517 | 0.067 | 0.938 | 0.045 |
| IL-8 +1633 | 4 | 823/1104 | 0.960 (0.836‒1.103) | | 0.924 (0.698‒1.223) | | 0.977 (0.789‒1.211) | | 0.958 (0.783‒1.172) | | 0.935 (0.726‒1.203) | |
|  |  |  | 0.166 | 0.564 | 0.197 | 0.580 | 0.569 | 0.835 | 0.304 | 0.675 | 0.372 | 0.600 |
| *IL-8 +781* |  |  |  | |  | |  | |  | |  | |
| Total | 19 | 3648/4428 | 0.928 (0.803‒1.073) | | 0.841 (0.613‒1.152) | | 1.008 (0.865‒1.175) | | 0.971 (0.816‒1.156) | | 0.842 (0.650‒1.089) | |
|  |  |  | <0.001 | 0.313 | <0.001 | 0.281 | 0.002 | 0.920 | 0.000 | 0.739 | <0.001 | 0.190 |
| *Ethnicity* |  |  |  | |  | |  | |  | |  | |
| Asian | 12 | 2723/3187 | 0.934 (0.813‒1.072) | | 0.799 (0.588‒1.084) | | 1.030 (0.870‒1.220) | | 0.984 (0.824‒1.175) | | 0.790 (0.613‒1.019) | |
|  |  |  | 0.001 | 0.329 | 0.001 | 0.150 | 0.019 | 0.728 | 0.004 | 0.860 | 0.007 | 0.069 |
| Caucasian | 3 | 466/723 | 1.189 (0.856‒1.653) | | 1.528 (0.773‒3.020) | | 1.225 (0.761‒1.973) | | 1.276 (0.746‒2.181) | | 1.472 (1.078‒2.009) | |
|  |  |  | 0.043 | 0.302 | 0.063 | 0.223 | 0.067 | 0.404 | 0.024 | 0.374 | 0.366 | 0.015 |
| African | 3 | 432/480 | 0.826 (0.406‒1.681) | | 0.809 (0.199‒3.297) | | 0.746 (0.461‒1.205) | | 0.750 (0.370‒1.521) | | 0.932 (0.282‒3.077) | |
|  |  |  | <0.001 | 0.598 | <0.001 | 0.767 | 0.070 | 0.231 | 0.001 | 0.426 | 0.001 | 0.907 |
| Mixed | 1 | 27/38 | 0.424 (0.208‒0.866) | | 0.164 (0.033‒0.810) | | 0.933 (0.271‒3.209) | | 0.536 (0.167‒1.718) | | 0.172 (0.044‒0.671) | |
|  |  |  | <0.001 | 0.018 | <0.001 | 0.027 | <0.001 | 0.913 | <0.001 | 0.294 | <0.001 | 0.011 |
| *Cancer type* |  |  |  | |  | |  | |  | |  | |
| Hepatocellular carcinoma | 4 | 902/1099 | 0.692 (0.467‒1.026) | | 0.437 (0.235‒0.812) | | 0.848(0.505‒1.424) | | 0.723 (0.417‒1.253) | | 0.499 (0.349‒0.711) | |
|  |  |  | <0.001 | 0.067 | 0.014 | 0.009 | <0.001 | 0.533 | <0.001 | 0.248 | 0.250 | <0.001 |
| Other cancer | 12 | 2302/2791 | 0.981 (0.822‒1.171) | | 0.957(0.635‒1.445) | | 1.032 (0.869‒1.225) | | 1.031 (0.842‒1.263) | | 0.946 (0.679‒1.319) | |
|  |  |  | <0.001 | 0.834 | <0.001 | 0.836 | 0.066 | 0.720 | 0.003 | 0.765 | <0.001 | 0.744 |
| Gastric cancer | 3 | 444/538 | 1.119 (0.931‒1.346) | | 1.267 (0.864‒1.859) | | 1.118 (0.845‒1.479) | | 1.148 (0.883‒1.494) | | 1.171 (0.826‒1.659) | |
|  |  |  | 0.456 | 0.232 | 0.675 | 0.226 | 0.411 | 0.434 | 0.368 | 0.303 | 0.859 | 0.375 |
| *Source of control* |  |  |  | |  | |  | |  | |  | |
| HB | 16 | 3217/3845 | 0.897 (0.761‒1.057) | | 0.764 (0.536‒1.090) | | 1.022 (0.855‒1.221) | | 0.958 (0.781‒1.175) | | 0.769 (0.580‒1.019) | |
|  |  |  | <0.001 | 0.194 | <0.001 | 0.138 | 0.001 | 0.814 | <0.001 | 0.681 | <0.001 | 0.067 |
| PB | 3 | 431/583 | 1.087 (0.846‒1.397) | | 1.336 (0.735‒2.428) | | 0.911 (0.693‒1.198) | | 1.002 (0.776‒1.294) | | 1.383 (0.799‒2.393) | |
|  |  |  | 0.191 | 0.514 | 0.147 | 0.343 | 0.919 | 0.505 | 0.512 | 0.987 | 0.170 | 0.247 |
| *Genotype* |  |  |  | |  | |  | |  | |  | |
| PCR-RFLP | 14 | 2787/3622 | 0.936 (0.792‒1.107) | | 0.841 (0.585‒1.209) | | 1.023 (0.849‒1.234) | | 0.980 (0.794‒1.209) | | 0.832 (0.619‒1.118) | |
|  |  |  | <0.001 | 0.441 | <0.001 | 0.351 | 0.001 | 0.809 | <0.001 | 0.849 | <0.001 | 0.222 |
| Other type | 5 | 861/806 | 0.892 (0.643‒1.238) | | 0.812 (0.387‒1.702) | | 0.972 (0.753‒1.256) | | 0.946 (0.679‒1.318) | | 0.872 (0.475‒1.601) | |
|  |  |  | 0.001 | 0.495 | 0.001 | 0.581 | 0.278 | 0.828 | 0.064 | 0.744 | 0.003 | 0.659 |
| *IL-8 -251* |  |  |  | |  | |  | |  | |  | |
| Total | 81 | 20295/25547 | 1.055 (1.000‒1.112) | | 1.098 (0.991‒1.215) | | 1.077 (0.997‒1.162) | | 1.086 (1.005‒1.173) | | 1.046 (0.966‒1.133) | |
|  |  |  | <0.001 | 0.049 | <0.001 | 0.073 | <0.001 | 0.059 | <0.001 | 0.037 | <0.001 | 0.268 |
| *Ethnicity* |  |  |  | |  | |  | |  | |  | |
| Caucasian | 25 | 7714/11634 | 0.989 (0.941‒1.039) | | 0.982 (0.891‒1.083) | | 0.948 (0.884‒1.017) | | 0.959 (0.896‒1.026) | | 1.016 (0.935‒1.103) | |
|  |  |  | 0.220 | 0.652 | 0.254 | 0.719 | 0.538 | 0.139 | 0.432 | 0.223 | 0.248 | 0.714 |
| Mixed | 9 | 847/1185 | 1.076 (0.876‒1.321) | | 1.093 (0.737‒1.619) | | 1.402 (1.041‒1.889) | | 1.288 (0.954‒1.740) | | 0.874 (0.646‒1.183) | |
|  |  |  | 0.023 | 0.483 | 0.047 | 0.659 | 0.086 | 0.026 | 0.042 | 0.099 | 0.122 | 0.384 |
| Asian | 41 | 10663/11708 | 1.102 (1.014‒1.197) | | 1.196 (1.015‒1.410) | | 1.121 (0.994‒1.264) | | 1.144 (1.014‒1.292) | | 1.114 (0.979‒1.267) | |
|  |  |  | <0.001 | 0.022 | <0.001 | 0.033 | <0.001 | 0.062 | <0.001 | 0.029 | <0.001 | 0.100 |
| African | 6 | 1071/1020 | 0.979 (0.660‒1.453) | | 0.947 (0.458‒1.960) | | 1.013 (0.657‒1.563) | | 0.981 (0.578‒1.663) | | 0.962 (0.577‒1.606) | |
|  |  |  | <0.001 | 0.917 | <0.001 | 0.884 | 0.004 | 0.952 | <0.001 | 0.942 | <0.001 | 0.883 |
| *Cancer type* |  |  |  | |  | |  | |  | |  | |
| Gastric cancer | 31 | 5861/8339 | 1.155 (1.057‒1.262) | | 1.297 (1.079‒1.560) | | 1.24 (1.085‒1.418) | | 1.252 (1.096‒1.430) | | 1.137 (0.986‒1.311) | |
|  |  |  | <0.001 | 0.002 | <0.001 | 0.006 | <0.001 | 0.002 | <0.001 | 0.001 | 0.001 | 0.077 |
| Colorectal cancer | 8 | 1889/2876 | 1.025 (0.907‒1.157) | | 1.036 (0.817‒1.314) | | 0.914 (0.793‒1.054) | | 0.950 (0.832‒1.085) | | 1.103 (0.892‒1.363) | |
|  |  |  | 0.056 | 0.697 | 0.061 | 0.770 | 0.871 | 0.217 | 0.544 | 0.447 | 0.032 | 0.365 |
| Other cancer | 8 | 1241/1871 | 1.098 (0.917‒1.314) | | 1.198 (0.844‒1.700) | | 1.042 (0.868‒1.250) | | 1.099 (0.896‒1.348) | | 1.119 (0.807‒1.55) | |
|  |  |  | 0.016 | 0.308 | 0.039 | 0.313 | 0.326 | 0.660 | 0.146 | 0.364 | 0.022 | 0.500 |
| Hepatocellular carcinoma | 6 | 1442/1399 | 1.023 (0.844‒1.241) | | 0.963 (0.690‒1.344) | | 1.209 (0.793‒1.842) | | 1.149 (0.779‒1.693) | | 0.828 (0.647‒1.06) | |
|  |  |  | 0.014 | 0.814 | 0.096 | 0.825 | <0.001 | 0.377 | < 0.001 | 0.484 | 0.255 | 0.134 |
| Oral cancer | 6 | 771/1545 | 0.951 (0.836‒1.082) | | 0.931 (0.713‒1.214) | | 0.902 (0.734‒1.108) | | 0.905 (0.745‒1.098) | | 0.987 (0.782‒1.244) | |
|  |  |  | 0.570 | 0.449 | 0.680 | 0.596 | 0.465 | 0.326 | 0.557 | 0.312 | 0.534 | 0.910 |
| Nasopharyngeal carcinoma | 4 | 721/920 | 1.079 (0.717‒1.623) | | 1.131(0.535‒2.392) | | 1.116 (0.703‒1.772) | | 1.124(0.658‒1.920) | | 1.075 (0.646‒1.789) | |
|  |  |  | <0.001 | 0.715 | <0.001 | 0.746 | 0.005 | 0.640 | <0.001 | 0.670 | 0.018 | 0.780 |
| Prostate cancer | 6 | 2211/2175 | 0.958 (0.862‒1.064) | | 0.936 (0.753‒1.163) | | 0.929 (0.770‒1.119) | | 0.927 (0.782‒1.099) | | 0.977 (0.816‒1.169) | |
|  |  |  | 0.200 | 0.422 | 0.168 | 0.549 | 0.121 | 0.436 | 0.143 | 0.383 | 0.210 | 0.798 |
| Breast cancer | 5 | 2459/2448 | 1.127 (0.823‒1.544) | | 1.299 (0.716‒2.358) | | 1.095 (0.761‒1.576) | | 1.159 (0.765‒1.757) | | 1.208 (0.791‒1.846) | |
|  |  |  | <0.001 | 0.422 | <0.001 | 0.389 | <0.001 | 0.625 | <0.001 | 0.486 | <0.001 | 0.382 |
| Lung cancer | 7 | 3700/3974 | 0.845 (0.717‒0.996) | | 0.758 (0.557‒1.031) | | 0.91 (0.794‒1.042) | | 0.843 (0.694‒1.024) | | 0.812 (0.632‒1.044) | |
|  |  |  | <0.001 | 0.045 | 0.004 | 0.077 | 0.292 | 0.173 | 0.028 | 0.086 | 0.003 | 0.105 |
| *Source of control* |  |  |  | |  | |  | |  | |  | |
| HB | 55 | 11613/14192 | 1.078 (1.005‒1.155) | | 1.126 (0.984‒1.289) | | 1.137 (1.024‒1.261) | | 1.139 (1.027‒1.264) | | 1.040 (0.936‒1.157) | |
|  |  |  | <0.001 | 0.035 | <0.001 | 0.085 | <0.001 | 0.016 | <0.001 | 0.014 | < 0.001 | 0.465 |
| PB | 26 | 8682/11355 | 1.017 (0.935‒1.105) | | 1.058 (0.903‒1.239) | | 0.986 (0.886‒1.097) | | 1.004 (0.895‒1.126) | | 1.057 (0.934‒1.196) | |
|  |  |  | <0.001 | 0.699 | < 0.001 | 0.487 | 0.001 | 0.792 | <0.001 | 0.947 | <0.001 | 0.380 |
| *Genetype* |  |  |  | |  | |  | |  | |  | |
| Taqman | 16 | 5338/7113 | 1.003 (0.906‒1.109) | | 1.03 (0.843‒1.258) | | 0.922 (0.808‒1.052) | | 0.951 (0.827‒1.094) | | 1.053 (0.906‒1.223) | |
|  |  |  | <0.001 | 0.961 | 0.001 | 0.772 | 0.037 | 0.228 | 0.005 | 0.485 | 0.004 | 0.503 |
| PCR-RFLP | 39 | 9505/10768 | 1.053 (0.966‒1.148) | | 1.083 (0.915‒1.282) | | 1.131 (1.000‒1.278) | | 1.120 (0.989‒1.268) | | 1.001 (0.876‒1.144) | |
|  |  |  | <0.001 | 0.237 | <0.001 | 0.352 | <0.001 | 0.050 | <0.001 | 0.075 | <0.001 | 0.986 |
| Other type | 13 | 2922/4632 | 1.103 (0.973‒1.25) | | 1.16 (0.927‒1.45) | | 1.127 (0.930‒1.367) | | 1.147 (0.949‒1.385) | | 1.096 (0.915‒1.313) | |
|  |  |  | 0.000 | 0.125 | 0.013 | 0.194 | 0.001 | 0.222 | <0.001 | 0.155 | 0.035 | 0.318 |
| ARMS-PCR | 4 | 651/836 | 0.974 (0.834‒1.137) | | 0.933 (0.684‒1.271) | | 1.030 (0.763‒1.391) | | 1.002 (0.792‒1.269) | | 0.923 (0.705‒1.207) | |
|  |  |  | 0.893 | 0.735 | 0.826 | 0.659 | 0.258 | 0.846 | 0.538 | 0.984 | 0.415 | 0.557 |
| PCR | 4 | 889/1087 | 0.955 (0.782‒1.166) | | 0.926 (0.637‒1.347) | | 0.992 (0.669‒1.469) | | 0.967 (0.663‒1.409) | | 0.939 (0.756‒1.167) | |
|  |  |  | 0.128 | 0.651 | 0.187 | 0.689 | 0.037 | 0.967 | 0.032 | 0.860 | 0.760 | 0.570 |
| AS-PCR | 5 | 990/1111 | 1.323 (1.074‒1.629) | | 1.697 (1.162‒2.478) | | 1.261 (1.000‒1.591) | | 1.381 (1.071‒1.78) | | 1.511 (1.066‒2.142) | |
|  |  |  | 0.055 | 0.008 | 0.089 | 0.006 | 0.338 | 0.050 | 0.204 | 0.013 | 0.050 | 0.020 |

*P*_h_, Value of *Q*-test for heterogeneity test; p, *Z*-test for the statistical significance of the OR; HB, Hospital-Based; PB, Population-Based; SOC, Source of Control.
